# Loss of Mitochondrial *Ndufs4* in Striatal Medium Spiny Neurons Mediates Progressive Motor Impairment in a Mouse Model of Leigh Syndrome

**DOI:** 10.3389/fnmol.2017.00265

**Published:** 2017-08-24

**Authors:** Byron Chen, Jessica Hui, Kelsey S. Montgomery, Alejandro Gella, Irene Bolea, Elisenda Sanz, Richard D. Palmiter, Albert Quintana

**Affiliations:** ^1^Department of Biochemistry, Howard Hughes Medical Institute, University of Washington Seattle, WA, United States; ^2^Center for Developmental Therapeutics and Center for Integrative Brain Research, Seattle Children’s Research Institute Seattle, WA, United States; ^3^Institut de Neurociències and Department of Cell Biology, Physiology and Immunology, Facultat de Medicina, Universitat Autònoma de Barcelona Bellaterra, Spain; ^4^Department of Pediatrics, University of Washington Seattle, WA, United States

**Keywords:** mitochondrial disease, mouse genetics, striatum, behavior, animal, medium spiny neuron

## Abstract

Inability of mitochondria to generate energy leads to severe and often fatal myoencephalopathies. Among these, Leigh syndrome (LS) is one of the most common childhood mitochondrial diseases; it is characterized by hypotonia, failure to thrive, respiratory insufficiency and progressive mental and motor dysfunction, leading to early death. Basal ganglia nuclei, including the striatum, are affected in LS patients. However, neither the identity of the affected cell types in the striatum nor their contribution to the disease has been established. Here, we used a mouse model of LS lacking *Ndufs4*, a mitochondrial complex I subunit, to confirm that loss of complex I, but not complex II, alters respiration in the striatum. To assess the role of striatal dysfunction in the pathology, we selectively inactivated *Ndufs4* in the striatal medium spiny neurons (MSNs), which account for over 95% of striatal neurons. Our results show that lack of *Ndufs4* in MSNs causes a non-fatal progressive motor impairment without affecting the cognitive function of mice. Furthermore, no inflammatory responses or neuronal loss were observed up to 6 months of age. Hence, complex I deficiency in MSNs contributes to the motor deficits observed in LS, but not to the neural degeneration, suggesting that other neuronal populations drive the plethora of clinical signs in LS.

## Introduction

Mutations in nuclear- or mitochondrial-encoded genes impairing energy metabolism lead to mitochondrial disease, a heterogeneous group of severe, and often fatal, pathologies affecting ~1:5000 live births. Leigh Syndrome (LS, OMIM 256,000) is the most common pediatric mitochondrial disease with a frequency of ~1:40,000 live births (Rahman et al., 1996), even though it is remarkably more common in some populations, ranging from 1:2000 up to 1:27 live births (Laberge et al., 2005; Finsterer and Zarrouk-Mahjoub, 2017). Mutations in over 75 different genes have been described to cause LS (Lake et al., 2016). Currently there is no effective treatment or cure to LS, with most therapeutic options being restricted to palliative approaches.

Although LS patients are highly heterogeneous clinically, the common signs and symptoms comprise failure to thrive, hypotonia, ataxia, lactic acidosis and encephalopathy (Finsterer, 2008a) that usually results in fatal respiratory failure at a young age (Arii and Tanabe, 2000; Ferrari et al., 2017). MRI studies on LS patients show the presence of characteristic symmetrical bilateral lesions, usually in the basal ganglia or brainstem (Arii and Tanabe, 2000). Even though these lesions have diagnostic value, the relative contribution to the phenotype of the cell type(s) affected by mitochondrial disease remains, for the most part, unknown.

We have characterized a mouse model of LS that lacks *Ndufs4*, a subunit of mitochondrial complex I. These animals (Ndufs4 knockout, KO mice) recapitulate most of the clinical features of human disease such as failure to thrive, hypotonia, ataxia and fatal respiratory failure by about post-natal day 50 (Quintana et al., 2010, 2012b). Patients harboring mutations in *NDUFS4* present lesions in basal ganglia and/or brainstem (Budde et al., 2003; Leshinsky-Silver et al., 2009). Accordingly, Ndufs4 KO mice develop CNS lesions in the in late stages of the disease, primarily in the brainstem and progressing to the striatum, a key member of the basal ganglia (Quintana et al., 2010; Quintana et al., 2012b). We have identified the role of brainstem lesions in the fatal breathing alterations observed in the Ndufs4 KO mice (Quintana et al., 2012b). However the contribution of the striatum to the phenotype, has not been determined.

The basal ganglia are a key relay in the motor system circuit between the motor cortex and thalamus, and are implicated in the control of motor coordination, voluntary movement, and cue-dependent learning, among other functions (Gerfen, 1992; Darvas and Palmiter, 2015). The striatum is a major component in this circuitry, integrating and relaying cortical inputs to other basal ganglia nuclei via GABAergic medium spiny neurons (MSNs), which constitute over 95% of striatal neurons (Gerfen, 1992). Thus we hypothesized that striatal MSN dysfunction may be responsible for the motor dysfunction observed in LS patients.

Here, we set out to establish the presence of primary striatal complex I-mediated mitochondrial dysfunction in Ndufs4 KO mice. Subsequently, to assess whether lack of *Ndufs4* in MSN is sufficient to elicit neurodegeneration and functional deficits or if it rather requires the cross-talk between different cell-types (Liu et al., 2015), we generated a mouse with conditional deletion of *Ndufs4* in striatal MSNs via Cre-mediated expression under the control of *Gpr88*, a pan-MSN promoter (Quintana et al., 2012a). We show that complex I deficiency in MSNs causes a mild, progressive motor impairment in mice, in the absence of inflammation or neuronal loss, and normal lifespan.

## Materials and Methods

### Animals

Mice were housed in a facility with a 12-h light:dark cycle. All animal experimentation was approved by the ethical committee at University of Washington (IACUC), Seattle Children’s Research Institute (IACUC), Universitat Autònoma de Barcelona (CEEAH) and Generalitat de Catalunya (DMAH). Two groups of animal lines were used in this study. To identify the presence of primary striatal deficits after *Ndufs4* deficiency, we used animals lacking *Ndufs4* constitutively (*Ndufs4*^−/−^, Ndufs4 KO) mice and wild-type (WT) mice (*Ndufs4*^+/+^) as controls. These mice were generated as described (Kruse et al., 2008; Quintana et al., 2010). To achieve conditional deletion of *Ndufs4* in striatum, mice expressing cre recombinase under the control of the *Gpr88* promoter (Quintana et al., 2012a) and heterozygous for *Ndufs4* (*Gpr88^cre/+^* ::*Ndufs4^Δ/+^*) were bred with mice with a floxed *Ndufs4* alleles (*Ndufs4^lox/lox^*). GPR88 is selective of striatal MSNs and does not target other cell types as striatal interneurons or astrocytes (Quintana et al., 2012a). Out of all the possible offspring, *Gpr88^cre/+^* ::*Ndufs4^Δ/lox^* mice have loss of Ndufs4 selectively in MSNs (MSN KO mice). *Mice with Gpr88^cre/+^* ::*Ndufs4^lox/+^* genotype with one functional Ndufs4 allele were used as controls (MSN CTL mice).

### Mitochondria Isolation

Mitochondria were isolated using Percoll density-gradient centrifugation as described (Wang et al., 2011) with some modifications. The striatum was dissected from the mouse brain between postnatal day 42 or 43. Briefly, the brain tissue was disrupted in 2 mL Isolation buffer A (IB_A_) using a 7-mL glass Dounce homogenizer. The tissue was gently passed by pestle A nine times and then pestle B eight times. The tissue supernatant with 80% Percoll was layered over 15% Percoll solution and then centrifuged at 16,200× *g* for 15 min at 4°C. 1.5-mL sucrose washing buffer (WB) was used to re-suspend the mitochondrial pellet. Following the final centrifugation, the pellets were re-suspended with 50 μL of WB with no bovine serum albumin (BSA) and combined into one tube. 1.5 mL of WB was added. An additional centrifugation (10,000× *g* for 5 min at 4°C) was performed and the final mitochondrial pellet was re-suspended in minimal (20 μL) WB with no BSA.

### Oxygen Consumption (Seahorse Assay)

Mitochondrial oxygen consumption rate (OCR) was measured over a 30-min time frame using the Seahorse Bioscience XF24 extracellular flux analyzer following the manufacturer’s instructions with some modifications (Johnson et al., 2013; Kayser et al., 2016). Before the day of the assay, the cartridge sensor was hydrated overnight with Seahorse Bioscience XF24 Calibration buffer at 37°C. On the day of the assay, 5 μg of mitochondria were added to assay media containing 500 mM malate and 500 mM pyruvate to assess complex I activity; alternatively, 2 μg of mitochondria were added to 500 mM succinate, a complex II substrate, and 2 mM rotenone, a complex I inhibitor. The two substrates independently measured complex I and complex II respiration. Real-time OCR (pmol/min/μg) was measured under basal condition and in response to mitochondrial inhibitors. The final value was corrected to account for protein loaded. Mitochondria were exposed to sequential injections of 40 mM ADP, 25 μg/mL oligomycin, 50 μM FCCP and 40 μg/μL antimycin A over 30 min. The final concentration of the inhibitors was reduced 10-fold in the assay mix. ADP initiated state III, oligomycin induced state IVo, FCCP induced an uncoupled state and antimycin A induced complete electron transport chain inhibition. Mitochondria isolated from *Ndufs4* KO brain tissue were compared to mitochondria isolated from a control WT tissue. The study was performed in four replicates with five technical replicates per condition. Technical replicates within a single experiment were averaged.

### Tissue Preparation

Mice were perfused with 4% paraformaldehyde (PFA) solution to preserve their brain tissue. After 24 h in PFA, the brains were cryopreserved in 30% sucrose PBS solution before freezing with solid CO_2_. Then frozen brains were sliced into 30-μm thick slices and suspended in a PBS solution in preparation for histological analyses. For western blotting, brains were dissected and snap-frozen in liquid nitrogen. Subsequently, samples were homogenized in 1× RIPA buffer prepared with protease and phosphatase inhibitors.

### Immunofluorescence

Tissue slices were rinsed in a phosphate buffered saline with 0.2% Tween (PBST) solution, non-specific binding was blocked with a 10% normal donkey serum (NDS)/PBST solution for 60 min at room temperature. Then tissue slices were incubated with the primary antibodies: Iba 1 (1:1000, WAKO), GFAP (1:2000, Sigma-Aldrich) in a 1% NDS/PBST solution overnight at 4°C. Subsequently, slices were again rinsed several times in PBST before applying fluorochrome-conjugated secondary antibodies (Jackson Immunoresearch) in a 1% NDS/PBST solution with a 1:200 dilution for 60 min, effectively tagging any primary antibodies bound to protein from the first staining with a fluorescent label.

### Western Blotting

Protein concentration was measured using the bicinchoninic acid (BCA) method (Thermo Scientific). Subsequently, 20 μg of cell lysate was denatured with sample buffer (62.5 mM Tris–HCl pH6.8, 2% (v/v) SDS, 50 mM DTT, 10% (v/v) glycerol), subjected to 4%–20% gradient SDS-PAGE, and transferred onto nitrocellulose membranes (BioRad). The membranes were blocked for 1 h with 5% (w/v) dried skimmed milk in TBS-T buffer (25 mM Tris, 150 mM NaCl, and 0.1% (v/v) Tween-20, pH 7.4), and incubated with the following primary antibodies: mouse anti-GFAP (Sigma-Aldrich; 1:50,000), rabbit anti-Iba1 (Wako; 1:10,000), mouse anti-β-actin (Sigma-Aldrich; 1:20,000), anti-NSE (1:1000, Dako), or anti-GAPDH (1:40000, GeneTex), at 4°C overnight. Then, membranes were incubated with the corresponding HRP-coupled secondary antibodies (Jackson Immunoresearch; 1:10,000) for 1 h at room temperature, and the bands were visualized using the Super Signal West Pico chemiluminescent substrate (Thermo Scientific). The immunoreactive signals were quantified using ImageJ software (National Institutes of Health, Bethesda, MD, USA). Iba1 and GFAP values were normalized to β-ACTIN values. NSE results, due to molecular weight, were normalized to GAPDH values.

### Behavioral Testing

#### Locomotion

To assess relative activity levels of the mice, mice were individually habituated for 48 h to the locomotion chambers (Columbus Instruments). Then spontaneous locomotion was assessed with *ad libitum* access to food and water for 48 h. Distance traveled was calculated by Optomax Software as described (Quintana et al., 2012a).

#### Motor Coordination

The rotarod was used to determine motor coordination and learning as described (Quintana et al., 2010). Mice were placed on an elevated accelerating rod (4–40 rpm in 4 min). Latency to fall was recorded for three sessions, one session/day, each consisting of three trials. Average values per session are shown.

#### Cue Learning

To asses cue learning, the water U-maze was employed (Quintana et al., 2012a). The test was carried out in a metal U maze with arms (50 cm, one black and one white) bent back toward the stem (45 cm). An escape platform, not visible from the end of stem, was present at the end of one of the two arms. Maze arms were alternated in a non-repetitive, pseudo-random sequence daily (10 trials per day, 3–5-min inter-trial interval). The percentage of correct trials was recorded. Mice remained in the maze until a correct turn was made to ensure an equal number of reinforced responses. An incorrect choice was recorded after a 20-s cutoff in the stem or if the mouse entered the wrong arm at any time.

The strategy-shifting procedure consisted of two phases. For phase 1, the mice had to learn a turn-based strategy for 3 days with the escape platform always on the same side of the ramp. For phase 2 (days 4–7), the platform was associated to the arm color (cue-based strategy).

### Statistics

Depending on the experimental design, Student’s *T*-test or 2-way analysis of variance (ANOVA) tests were performed using Graphpad Prism software, *p* < 0.05 was considered as significant. Data are shown as Mean ± SEM.

## Results

### Lack of *Ndufs4* Impairs Striatal Complex I-Mediated Respiration

**Figure 1:**
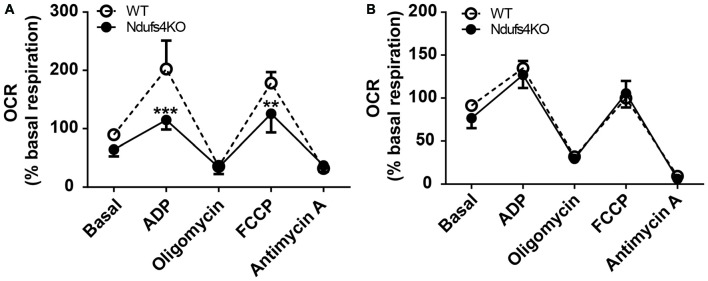
Complex I-mediated respiratory alterations in Ndufs4 knockout (KO) mice. **(A)** Complex I-dependent respiration (using pyruvate/malate as substrates) and **(B)** complex II-mediated respiration (using succinate as substrate) in striata from Ndufs4 KO mice (*n* = 5) compared to wild-type (WT) mice (*n* = 5). Basal: 500 mM malate and 500 mM pyruvate (CI) or 500 mM succinate and 2 mM of the CI inhibitor rotenone (CII). ADP (40 mM) initiated state III, oligomycin (25 μg/mL) induced state IVo, FCCP (50 μM) induced an uncoupled state and antimycin A (40 μg/μL) induced complete electron transport chain inhibition. **p < 0.01, ***p < 0.001 vs. WT. Data are shown as mean ± SEM.

Lesions in the striatum have been observed in humans harboring *NDUFS4* mutations (Budde et al., 2003) as well as in Ndufs4 KO mice (Quintana et al., 2012b). However, the existence of constitutive mitochondrial alterations in striata of Ndufs4 KO mice had not been assessed. To identify primary striatal mitochondrial defects, both complex I (CI)- and complex II (CII)-dependent mitochondrial respiratory rates were determined by performing extracellular flux analysis in purified striatal mitochondrial preparations from Ndufs4 KO and control mice (Figure 1). Striatal mitochondria from Ndufs4KO mice presented reduced OCR after administration of the CI substrates pyruvate and malate and ADP (state 3 respiration) and reduced maximal respiratory capacity after administration of the ETC uncoupler FCCP (Figure 1A). On the other hand, normal state 3 and maximal respiratory capacity OCR were obtained when the CII substrate succinate together with CI-inhibitor rotenone were provided (Figure 1B), suggesting a selective CI-mediated dysfunction in the striata of Ndufs4KO.

### Selective *Ndufs4* Deletion in Striatal MSNs Does Not Affect Lifespan or Body Weight

**Figure 2:**
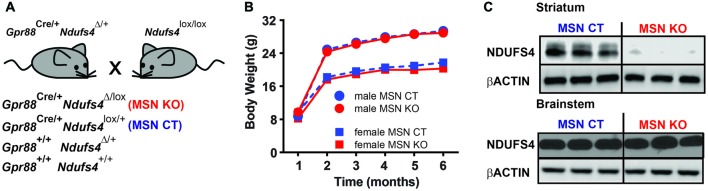
Generation of a mouse line lacking Ndufs4 in the striatal medium spiny neurons (MSNs). **(A)** Breeding strategy to generate animals with conditional *Ndufs4* deletion in striatal MSNs (MSN KO) and their genetic controls (MSN CTL). **(B)** Body weight curves for MSN KO mice (*n* = 5 males, *n* = 6 females) and MSN CTL (*n* = 14 males, *n* = 11 females). **(C)** Representative western blot analyses of total Ndufs4 and β-Actin (loading control) in the striatum and brainstem of MSN KO and CTL mice (*n* = 3). Data are shown as mean ± SEM.

To assess the specific contribution of striatal deficiency to the fatal phenotype observed in animals lacking *Ndufs4* (Quintana et al., 2010) we generated a new mouse line lacking *Gpr88* expression in the striatum. To achieve this, we conditionally ablated of *Ndufs4* in striatum by driving selective Cre recombinase expression under the *Gpr88* promoter (Quintana et al., 2012a) in animals with floxed *Ndufs4* alleles (Quintana et al., 2012b). *Gpr88* expression is selectively expressed in medium spiny neurons (Quintana et al., 2012a), which account for over 95% of striatal neuronal population (Gerfen, 1992). To minimize ectopic recombination during embryogenesis, Cre-expressing male mice heterozygous for *Ndufs4* gene were crossed with females carrying two floxed *Ndufs4* alleles. With this strategy animals lacking striatal *Ndufs4* expression (MSN KO) and their controls (MSN CTL, heterozygous for *Ndufs4*) were obtained (Figure 2A). MSN KO mice were born at the expected Mendelian frequencies. No differences in lifespan or body weight were observed in either male or female MSN KO mice compared to their controls up to 6 months of age (Figure 2B). Western blot analyses confirmed a selective decrease in NDUFS4 abundance in the striata of MSN KO mice compared to controls (Figure 2C).

### Progressive Locomotor Deficits after Striatal MSN *Ndufs4* Deletion

**Figure 3:**
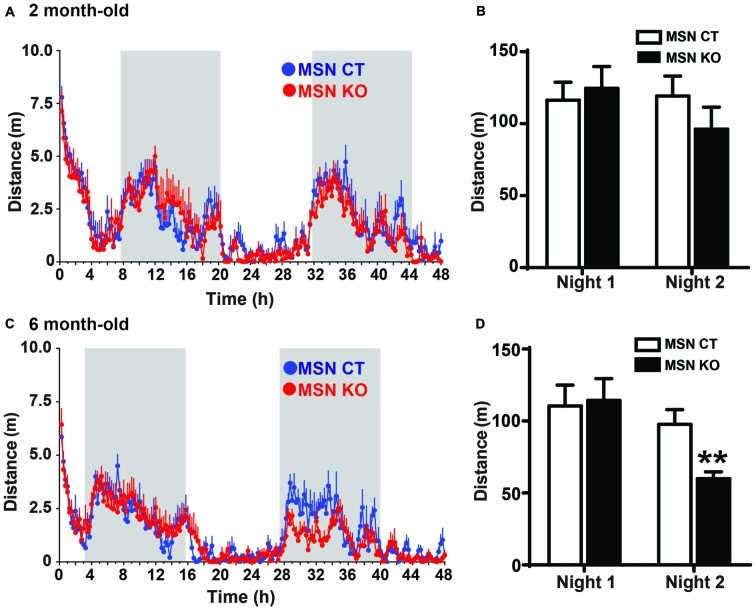
Reduced activity in MSN KO mice. Spontaneous activity was recorded for 48 h. Activity pattern **(A)** and quantification of nocturnal activity **(B)** of 2-month-old MSN KO (*n* = 6) and MSN CTL mice (*n* = 6). **(C)** Activity pattern and **(D)** quantification of nocturnal activity of 6-month-old MSN CTL (*n* = 7) and MSN KO mice (*n* = 8); **p < 0.01. Data are shown as mean ± SEM.

The striatum is a critical component of motor systems, influencing movement through cortical and thalamic connections (Alexander and Crutcher, 1990; Hoover and Strick, 1993; Kravitz et al., 2010). To assess locomotor patterns of MSN KO and control mice, their home-cage activity was monitored for 48 h. At 2 months of age the activity of MSN KO mice was the same as controls (Figures 3A,B). However, when they were tested at 6 months of age, MSN KO mice had significantly reduced locomotor activity on the second night of the test, when animals are fully habituated to the new environment (Figures 3C,D).

**Figure 4:**
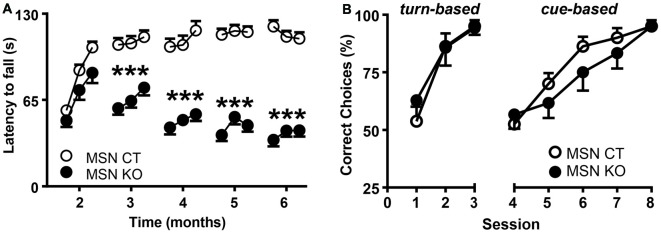
Motor alterations but intact associative learning in MSN KO mice. **(A)** Rotarod performance of MSN KO mice (*n* = 13) and MSN CTL mice (*n* = 26). **(B)** Water U-maze performance of 6-month-old MSN KO mice (*n* = 7) and MSN CTL mice (*n* = 8). Animals were trained for 3 days on a turn-based escape procedure and subsequently forced to change to a cue-based escape strategy. ***p < 0.001 vs. Ndufs4cCT^*Gpr88*^. Data are shown as mean ± SEM.

To assess motor coordination, MSN KO and MSN CTL mice were tested monthly on an accelerating rod and their latencies to fall were recorded (Figure 4A). MSN KO mice performed the same as controls at 2 months of age, but their performance progressively worsened between 3 and 6 months of age, revealing a severe deficit in motor coordination and learning (Figure 4A).

### Associative Learning Is Unaffected after Loss of *Ndufs4* in Striatal MSNs

Striatal function is necessary for associative learning (Packard and Knowlton, 2002) and contributes to cognitive flexibility processes (Darvas and Palmiter, 2009, 2011, 2015; Smith and Graybiel, 2014) such as adapting to strategy shifting. Therefore, we used a water-based, U-maze procedure (Quintana et al., 2012a) to test the ability of 6-month-old mice to identify the correct strategy to find an escape platform at the end of one of the maze arms. During the first 3 days, mice were trained daily to learn a turn-based strategy. In this paradigm, both MSN KO and control mice presented a similar percentage of correct choices and learning rate (Figure 4B). Subsequently, mice were forced to shift to a cue-based strategy, by associating the platform to the color of the arms, for five daily sessions. After the shift, there was a decrease in the number of correct choices, improving with further training. However, no differences between genotypes were observed (Figure 4B).

### Loss of *Ndufs4* in Striatal MSNs Does Not Induce Reactive Gliosis or Neuronal Loss

**Figure 5:**
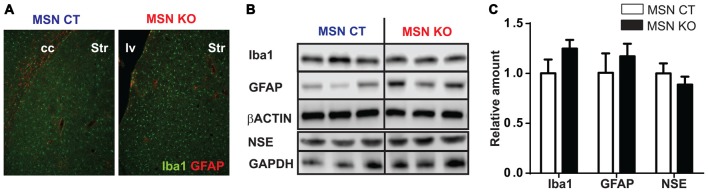
Lack of reactive gliosis in the striatum or neuronal loss after *Ndufs4* ablation in MSNs. **(A)** Representative GFAP (red) and Iba1 (green) immunofluorescence in striatum (Str) of MSN KO and MSN CTL mice. cc: corpus callosum. lv: lateral ventricle. **(B,C)** Representative western blot analyses of GFAP, Iba1, NSE and β-actin and GAPDH (loading controls) and quantification **(C)** in the striatum and brainstem of MSN KO and MSN CTL mice (*n* = 3–9). Data are shown as mean ± SEM.

Reactive gliosis is one of the hallmarks of LS (Finsterer, 2008a). To assess the presence of astroglial and microglial in MSN KO mice we performed immunofluorescence labeling against GFAP and Iba-1, respectively, in striata of 6-month-old MSN KO and control mice. Morphologically, both genotypes presented non-hypertrophic astrocytes and ramified microglial cells, suggestive of a nonreactive status (Raivich et al., 1999; Figure 5A). Total abundance of GFAP and Iba1 was not different in the striata of MSN KO and control mice as assessed by Western Blot (Figures 5B,C).

To assess neuronal loss, neuron specific enolase (NSE) levels were quantified (Scarna et al., 1982; Ding et al., 2000). Total striatal NSE abundance was not altered in the striata of MSN KO mice compared to MSN control as assessed by Western Blot (Figures 5B,C).

## Discussion

One of the main clinical hallmarks of LS is the presence of symmetrical brain lesions, typically in the brainstem or in the basal ganglia (Rahman et al., 1996; Arii and Tanabe, 2000; Finsterer, 2008a). While brainstem affectation is overtly evident in Ndufs4 KO mice, basal ganglia are relatively spared histopathologically in these mice (Quintana et al., 2010). However, our previous work reveals lesions in the striatum in Ndufs4 KO mice in which brainstem lesions were suppressed (Quintana et al., 2012b). Hence, we decided to explore the contribution of selective striatal mitochondrial dysfunction to the phenotype of these mice.

In this study, we identify the existence of an intrinsic deficit in Complex I-mediated mitochondrial respiration in the striata of Ndufs4 KO mice, suggesting that progressive impairment in this area may be conducive to the appearance of lesions. Impairment in CI-mediated mitochondrial respiration had been reported in different tissues both in human patients and mice lacking functional NDUFS4 expression (Budde et al., 2003; Kruse et al., 2008; Leong et al., 2012; Kayser et al., 2016; Ortigoza-Escobar et al., 2016). A recent report has shown region-specific alterations in the respiratory capacities of Ndufs4 KO mice (Kayser et al., 2016), correlating with the histopathological features. Hence, it is likely that the delay in appearance of striatal lesions is the result of a milder impairment in respiratory capacity.

To assess the specific role of striatal mitochondrial CI-dysfunction, we conditionally ablated *Ndufs4* in medium spiny neurons (MSN KO mice). Accordingly, striatal NDUFS4 levels were markedly reduced in in these mice. However, this reduction was not associated with reduced body weight or premature death, hallmarks of the disease in mice lacking NDUFS4 protein in all cells or just in the central nervous system (Kruse et al., 2008; Quintana et al., 2010) or LS patients (Rahman et al., 1996; Finsterer, 2008a), suggesting that these clinical signs are not mediated by MSN dysfunction. In this regard, we had previously shown that Ndufs4 KO mice presented lesions in the brainstem, associated with breathing alterations and death (Quintana et al., 2010, 2012b). Accordingly, brainstem lesions have been associated with central respiratory deficits and poor prognosis in human patients (Arii and Tanabe, 2000).

Behaviorally, MSN KO mice presented a progressive motor impairment, with reduced spontaneous locomotion and worsening of motor coordination as assessed by the rotarod test. Loss of already acquired psychomotor skills (as observed in the rotarod test) is one of the first clinical signs of LS patients (Rahman et al., 1996; Finsterer, 2008a). The striatum plays a pivotal role in movement control by integrating cortical, thalamic and dopaminergic inputs (Alexander and Crutcher, 1990; Gerfen, 1992; Hoover and Strick, 1993). MSNs, the only projection neurons in the striatum, consist of two different subpopulations (defined by the expression of either dopamine D1 or D2 receptors) that modulate movement by two opposing output pathways, the striatonigral and the striatopallidal, respectively (Gerfen, 1992; Kravitz et al., 2010; Lanciego et al., 2012). In this study Cre recombinase was driven by the *Gpr88* promoter, which expressed all MSNs but does not target striatal interneurons or astrocytes (Quintana et al., 2012a). Hence, our results suggest that striatal mitochondrial dysfunction in MSNs is sufficient to recapitulate the motor deficits of the human disease.

Cognitive impairment is frequently observed in LS patients (Finsterer, 2008b). Cortico-striatal basal ganglia pathways are necessary to properly perform several cognitive tasks (Quintana et al., 2012a; Darvas and Palmiter, 2015). However, MSN KO mice performed as well as controls in the U-maze test, suggesting that mitochondrial dysfunction in MSNs is not sufficient to impair associative learning or cognitive flexibility. It is likely that other brain areas, such as cortex, compensate for striatal deficits. Alternatively, MSN function may be sufficiently maintained to allow for correct striatal processing of cognitive processes.

We did not detect any signs of reactive gliosis, as assessed by immunofluorescence and western blot analyses for astrocyte (GFAP) or microglial (IBA1) markers. Accordingly, it has recently been described that mitochondrial dysfunction in Ndufs4 KO mice is present even in brain areas not associated to inflammation and histological alterations (Kayser et al., 2016). Furthermore, no neuronal loss was observed in MSN KO up to 6 months of age, in contrast to our previous results (Quintana et al., 2010, 2012b). These results may suggest that a striatal cell population other than MSNs contributes to the appearance of inflammation and neuronal loss. Alternatively, they may result from defects in brain regions that project axons to the striatum. In this regard, it has been described that axonal degeneration may lead to inflammation and lesion formation in the terminal fields (Coleman, 2005).

A key remaining aspect is identifying the mechanism underlying the loss of motor function without overt inflammation of neurodegeneration. Several possibilities may explain these findings. First, while NSE determination has been used an indirect correlate of neurodegeneration (Scarna et al., 1982; Ding et al., 2000) it does not possess the resolution and the sensitivity of stereological cell counts to detect neuronal loss. Further studies aimed at a detailed histological characterization of MSN KO striata are warranted. Second, the fact that MSN KO are heterozygous for *Ndufs4* in all cell types not expressing Cre recombinase may initiate compensatory mechanisms leading to mitochondrial preconditioning, which has been proven neuroprotective in several paradigms (Correia et al., 2010). Along the same lines, another possibility is that neurodegeneration may be the result of a complex cross-talk between cell-types rather than a cell-autonomous mechanism. Our previous results have pointed to the requirement of neuronal-astrocyte cross-talk to elicit neurodegeneration in Ndufs4KO mice (Liu et al., 2015). However, in MSN KO mice astrocytic mitochondrial function is likely spared. Finally, a recent study shows that *Ndufs4* deletion in dopaminergic neurons causes Parkinson’s disease-like non-motor symptoms without neuronal loss, likely due to reduced dopamine brain levels (Choi et al., 2017). Hence, it is feasible that even though not sufficient to elicit neurodegeneration, lack of *Ndufs4* may alter MSN function leading to behavioral deficits. Further studies will be directed at characterizing in more detail MSN physiology after *Ndufs4* ablation.

In conclusion, we have identified that *Ndufs4* deficiency leads to a reduction in the respiratory capacity in the striatum. Furthermore, specific deletion of this gene in MSNs leads to a progressive motor impairment, with absence of overt reactive gliosis, inflammation or neuronal loss. These results underscore the contribution of basal ganglia alterations to the motor phenotype observed in LS. Currently, there is no effective treatment for LS, although several potential therapies have been proposed (Johnson et al., 2013; Jain et al., 2016; Ferrari et al., 2017). Future investigations are needed to establish the relative contribution of susceptible brain regions to the pathology, which may help identify the therapeutic potential of new treatments.

## Author Contributions

BC, JH, KSM, IB, AG, ES and AQ performed research, analyzed the data and wrote the article. RDP and AQ provided reagents, designed experiments, coordinated the work and wrote and edited the manuscript. BC and JH contributed equally to this work.

## Conflict of Interest Statement

The authors declare that the research was conducted in the absence of any commercial or financial relationships that could be construed as a potential conflict of interest.

## References

[B1] Alexander G. E. Crutcher M. D. (1990). Functional architecture of basal ganglia circuits: neural substrates of parallel processing. Trends Neurosci. 13, 266–271. 10.1016/0166-2236(90)90107-l 1695401

[B2] Arii J. Tanabe Y. (2000). Leigh syndrome: serial MR imaging and clinical follow-up. Am. J. Neuroradiol. 21, 1502–1509. 11003287 PMC7974045

[B3] Budde S. M. van den Heuvel L. P. Smeets R. J. Skladal D. Mayr J. A. Boelen C.. (2003). Clinical heterogeneity in patients with mutations in the NDUFS4 gene of mitochondrial complex I. J. Inherit. Metab. Dis. 26, 813–815. 10.1023/b:boli.0000010003.14113.af 14765537

[B4] Choi W.-S. Kim H.-W. Tronche F. Palmiter R. D. Storm D. R. Xia Z. (2017). Conditional deletion of Ndufs4 in dopaminergic neurons promotes Parkinson’s disease-like non-motor symptoms without loss of dopamine neurons. Sci. Rep. 7:44989. 10.1038/srep44989 28327638 PMC5361188

[B5] Coleman M. (2005). Axon degeneration mechanisms: commonality amid diversity. Nat. Rev. Neurosci. 6, 889–898. 10.1038/nrn1788 16224497

[B6] Correia S. C. Carvalho C. Cardoso S. Santos R. X. Santos M. S. Oliveira C. R.. (2010). Mitochondrial preconditioning: a potential neuroprotective strategy. Front. Aging Neurosci. 2:138. 10.3389/fnagi.2010.00138 20838473 PMC2936931

[B7] Darvas M. Palmiter R. D. (2009). Restriction of dopamine signaling to the dorsolateral striatum is sufficient for many cognitive behaviors. Proc. Natl. Acad. Sci. U S A 106, 14664–14669. 10.1073/pnas.0907299106 19667174 PMC2731845

[B8] Darvas M. Palmiter R. D. (2011). Contributions of striatal dopamine signaling to the modulation of cognitive flexibility. Biol. Psychiatry 69, 704–707. 10.1016/j.biopsych.2010.09.033 21074144 PMC3120097

[B9] Darvas M. Palmiter R. D. (2015). Specific contributions of N-methyl-D-aspartate receptors in the dorsal striatum to cognitive flexibility. Neuroscience 284, 934–942. 10.1016/j.neuroscience.2014.11.011 25446363 PMC4267923

[B10] Ding M. Haglid K. G. Hamberger A. (2000). Quantitative immunochemistry on neuronal loss, reactive gliosis and BBB damage in cortex/striatum and hippocampus/amygdala after systemic kainic acid administration. Neurochem. Int. 36, 313–318. 10.1016/s0197-0186(99)00139-4 10732998

[B11] Ferrari M. Jain I. H. Goldberger O. Rezoagli E. Thoonen R. Chen K. H.. (2017). Hypoxia treatment reverses neurodegenerative disease in a mouse model of Leigh syndrome. Proc. Natl. Acad. Sci. U S A 114, E4241–E4250. 10.1073/pnas.1621511114 28483998 PMC5448167

[B12] Finsterer J. (2008a). Leigh and Leigh-like syndrome in children and adults. Pediatr. Neurol. 39, 223–235. 10.1016/j.pediatrneurol.2008.07.013 18805359

[B13] Finsterer J. (2008b). Cognitive decline as a manifestation of mitochondrial disorders (mitochondrial dementia). J. Neurol. Sci. 272, 20–33. 10.1016/j.jns.2008.05.011 18572195

[B14] Finsterer J. Zarrouk-Mahjoub S. (2017). NDUFS4-related Leigh syndrome in Hutterites. Am. J. Med. Genet. A 173, 1450–1451. 10.1002/ajmg.a.38225 28371352

[B15] Gerfen C. R. (1992). The neostriatal mosaic: multiple levels of compartmental organization in the basal ganglia. Annu. Rev. Neurosci. 15, 285–320. 10.1146/annurev.neuro.15.1.285 1575444

[B16] Hoover J. E. Strick P. L. (1993). Multiple output channels in the basal ganglia. Science 259, 819–821. 10.1126/science.7679223 7679223

[B17] Jain I. H. Zazzeron L. Goli R. Alexa K. Schatzman-Bone S. Dhillon H.. (2016). Hypoxia as a therapy for mitochondrial disease. Science 352, 54–61. 10.1126/science.aad9642 26917594 PMC4860742

[B18] Johnson S. C. Yanos M. E. Kayser E. B. Quintana A. Sangesland M. Castanza A.. (2013). mTOR inhibition alleviates mitochondrial disease in a mouse model of Leigh syndrome. Science 342, 1524–1528. 10.1126/science.1244360 24231806 PMC4055856

[B19] Kayser E. B. Sedensky M. M. Morgan P. G. (2016). Region-specific defects of respiratory capacities in the *Ndufs4*(KO) mouse brain. PLoS One 11:e0148219. 10.1371/journal.pone.0148219 26824698 PMC4732614

[B20] Kravitz A. V. Freeze B. S. Parker P. R. Kay K. Thwin M. T. Deisseroth K.. (2010). Regulation of parkinsonian motor behaviours by optogenetic control of basal ganglia circuitry. Nature 466, 622–626. 10.1038/nature09159 20613723 PMC3552484

[B21] Kruse S. E. Watt W. C. Marcinek D. J. Kapur R. P. Schenkman K. A. Palmiter R. D. (2008). Mice with mitochondrial complex I deficiency develop a fatal encephalomyopathy. Cell Metab. 7, 312–320. 10.1016/j.cmet.2008.02.004 18396137 PMC2593686

[B22] Laberge A. M. Michaud J. Richter A. Lemyre E. Lambert M. Brais B.. (2005). Population history and its impact on medical genetics in Quebec. Clin. Genet. 68, 287–301. 10.1111/j.1399-0004.2005.00497.x 16143014

[B23] Lake N. J. Compton A. G. Rahman S. Thorburn D. R. (2016). Leigh syndrome: one disorder, more than 75 monogenic causes. Ann. Neurol. 79, 190–203. 10.1002/ana.24551 26506407

[B24] Lanciego J. L. Luquin N. Obeso J. A. (2012). Functional neuroanatomy of the basal ganglia. Cold Spring Harb. Perspect. Med. 2:a009621. 10.1101/cshperspect.a009621 23071379 PMC3543080

[B25] Leong D. W. Komen J. C. Hewitt C. A. Arnaud E. McKenzie M. Phipson B.. (2012). Proteomic and metabolomic analyses of mitochondrial complex I-deficient mouse model generated by spontaneous B2 short interspersed nuclear element (SINE) insertion into NADH dehydrogenase (ubiquinone) Fe-S protein 4 (*Ndufs4*) gene. J. Biol. Chem. 287, 20652–20663. 10.1074/jbc.M111.327601 22535952 PMC3370248

[B26] Leshinsky-Silver E. Lebre A. S. Minai L. Saada A. Steffann J. Cohen S.. (2009). NDUFS4 mutations cause Leigh syndrome with predominant brainstem involvement. Mol. Genet. Metab. 97, 185–189. 10.1016/j.ymgme.2009.03.002 19364667

[B27] Liu L. Zhang K. Sandoval H. Yamamoto S. Jaiswal M. Sanz E.. (2015). Glial lipid droplets and ROS induced by mitochondrial defects promote neurodegeneration. Cell 160, 177–190. 10.1016/j.cell.2014.12.019 25594180 PMC4377295

[B28] Ortigoza-Escobar J. D. Oyarzabal A. Montero R. Artuch R. Jou C. Jiménez C.. (2016). Ndufs4 related Leigh syndrome: a case report and review of the literature. Mitochondrion 28, 73–78. 10.1016/j.mito.2016.04.001 27079373

[B29] Packard M. G. Knowlton B. J. (2002). Learning and memory functions of the basal ganglia. Annu. Rev. Neurosci. 25, 563–593. 10.1146/annurev.neuro.25.112701.142937 12052921

[B30] Quintana A. Kruse S. E. Kapur R. P. Sanz E. Palmiter R. D. (2010). Complex I deficiency due to loss of Ndufs4 in the brain results in progressive encephalopathy resembling Leigh syndrome. Proc. Natl. Acad. Sci. U S A 107, 10996–11001. 10.1073/pnas.1006214107 20534480 PMC2890717

[B31] Quintana A. Sanz E. Wang W. Storey G. P. Güler A. D. Wanat M. J.. (2012a). Lack of GPR88 enhances medium spiny neuron activity and alters motor- and cue-dependent behaviors. Nat. Neurosci. 15, 1547–1555. 10.1038/nn.3239 23064379 PMC3483418

[B32] Quintana A. Zanella S. Koch H. Kruse S. E. Lee D. Ramirez J. M.. (2012b). Fatal breathing dysfunction in a mouse model of Leigh syndrome. J. Clin. Invest. 122, 2359–2368. 10.1172/JCI62923 22653057 PMC3387817

[B33] Rahman S. Blok R. B. Dahl H. H. Danks D. M. Kirby D. M. Chow C. W.. (1996). Leigh syndrome: clinical features and biochemical and DNA abnormalities. Ann. Neurol. 39, 343–351. 10.1002/ana.410390311 8602753

[B34] Raivich G. Bohatschek M. Kloss C. U. Werner A. Jones L. L. Kreutzberg G. W. (1999). Neuroglial activation repertoire in the injured brain: graded response, molecular mechanisms and cues to physiological function. Brain Res. Rev. 30, 77–105. 10.1016/s0165-0173(99)00007-7 10407127

[B35] Scarna H. Delafosse B. Steinberg R. Debilly G. Mandrand B. Keller A.. (1982). Neuron-specific enolase as a marker of neuronal lesions during various comas in man. Neurochem. Int. 4, 405–411. 10.1016/0197-0186(82)90083-3 20487894

[B36] Smith K. S. Graybiel A. M. (2014). Investigating habits: strategies, technologies and models. Front. Behav. Neurosci. 8:39. 10.3389/fnbeh.2014.00039 24574988 PMC3921576

[B37] Wang X. Leverin A.-L. Han W. Zhu C. Johansson B. R. Jacotot E.. (2011). Isolation of brain mitochondria from neonatal mice. J. Neurochem. 119, 1253–1261. 10.1111/j.1471-4159.2011.07525.x 21985402 PMC3532608

